# Genetic analysis of sinonasal undifferentiated carcinoma discovers recurrent SWI/SNF alterations and a novel PGAP3-SRPK1 fusion gene

**DOI:** 10.1186/s12885-021-08370-x

**Published:** 2021-05-29

**Authors:** Molly E. Heft Neal, Andrew C. Birkeland, Apurva D. Bhangale, Jingyi Zhai, Aditi Kulkarni, Susan K. Foltin, Brittany M. Jewell, Megan L. Ludwig, Lisa Pinatti, Hui Jiang, Jonathan B. McHugh, Lawence Marentette, Erin L. McKean, J. Chad Brenner

**Affiliations:** 1 Department of Otolaryngology – Head and Neck Surgery, University of Michigan, 1150 E. Medical Center Dr., 9301B MSRB3, Ann Arbor, MI 48109-0602 USA; 2 Department of Biostatistics, University of Michigan, Ann Arbor, MI USA; 3 Program in Cellular and Molecular Biology, University of Michigan, 1150 E. Medical Center Dr., 9301B MSRB3, Ann Arbor, MI 48109-0602 USA; 4 Program in Cancer Biology, University of Michigan, Ann Arbor, MI USA; 5 Rogel Cancer Center, University of Michigan Medical School, 1150 E. Medical Center Dr., 9301B MSRB3, Ann Arbor, MI 48109-0602 USA; 6 Department of Pathology, University of Michigan, Ann Arbor, MI USA; 7 Department of Pharmacology, University of Michigan, 1150 E. Medical Center Dr., 9301B MSRB3, Ann Arbor, MI 48109-0602 USA

**Keywords:** SNUC, SWI/SNF, SMARCA

## Abstract

**Background:**

Sinonasal Undifferentiated Carcinoma (SNUC) is a rare and aggressive skull base tumor with poor survival and limited treatment options. To date, targeted sequencing studies have identified *IDH2* and *SMARCB1* as potential driver alterations, but the molecular alterations found in *SMARCB1* wild type tumors are unknown.

**Methods:**

We evaluated survival outcomes in a cohort of 46 SNUC patients treated at an NCI designated cancer center and identify clinical and disease variables associated with survival on Kaplan-Meier and Cox multivariate survival analysis. We performed exome sequencing to characterize a series of SNUC tumors (*n* = 5) and cell line (MDA8788–6) to identify high confidence mutations, copy number alterations, microsatellite instability, and fusions. Knockdown studies using siRNA were utilized for validation of a novel *PGAP3-SRPK1* gene fusion.

**Results:**

Overall survival analysis revealed no significant difference in outcomes between patients treated with surgery +/− CRT and CRT alone. Tobacco use was the only significant predictor of survival. We also confirmed previously published findings on *IDH* and *SMARC* family mutations and identified novel recurrent aberrations in the *JAK/STAT* and *PI3K* pathways. We also validated a novel *PGAP3*-*SRPK1* gene fusion in the SNUC cell line, and show that knockdown of the fusion is negatively associated with *EGFR*, *E2F* and *MYC* signaling.

**Conclusion:**

Collectively, these data demonstrate recurrent alterations in the *SWI/SNF* family as well as *IDH, JAK/STAT, and PI3K pathways* and discover a novel fusion gene (*PGAP3-SRPK1*). These data aim to improve understanding of possible driver mutations and guide future therapeutic strategies for this disease.

**Supplementary Information:**

The online version contains supplementary material available at 10.1186/s12885-021-08370-x.

## Background

Sinonasal Undifferentiated Carcinoma (SNUC) is a highly aggressive disease involving the anterior skull base, nasal cavity and paranasal sinuses. It is a rare tumor, with only a few hundred cases in the literature [1]. Patients usually present at an advanced stage, and have poor outcomes [2, 3], with two-year overall survival rates as low as 25% in some cohorts [1, 4–9]. Validated prognostic factors are limited to traditional clinical variables (overall stage, high grade, and poor differentiation), and no additional data on possible informative biomarkers is currently in clinical use [10]. Current treatment modalities including surgery, radiation, and systemic chemotherapy alone or in combination with radiation (CRT) have poor outcomes and carry significant toxicity to patients [11–13]. A recent study reveals improved survival with chemoselection paradigms, with five-year disease free survival rates of 59% in the total cohort and rates as high as 81% in responders [14]. However, despite these promising results, patients who did not show initial response to induction chemotherapy had a 0 and 39% five-year DSS when treated with CRT and surgery +/− CRT respectively. These results indicate the urgent need for novel therapeutics particularly for this subset of patients with aggressive disease. Importantly, there have been no novel or targeted agents introduced for SNUC treatment since its initial identification, which is partially due to a limited investigation into the underlying genetics defining SNUC pathogenesis.

To date, only a few case reports describing mutations associated with disease pathogenesis have been published. The most commonly reported mutations include *IDH2* and *SMARCB1* which have been identified in small case series via traditional sequencing approaches or targeted sequencing panels [15–17]. There have been additional case reports of potentially actionable mutations in isolated SNUCs including *ERBB2* and *FGFR1* [18, 19], but previous efforts have been limited in their scope of sequencing [4] and currently there have been no comprehensive whole exome or genome sequencing studies performed on SNUCs.

As such, this rare, devastating disease has limited treatment options currently available and characterizing genomic profiles of SNUCs may have significant benefit for the future development of rational therapeutic strategies. By understanding the genomic architecture behind this disease process, we may also begin to identify prognostic biomarkers that help identify the patients that fail current treatment paradigms. Here, we provide survival data from 46 patients treated at our tertiary referral center and report the first whole exome sequences profiling the mutational landscape of SNUCs.

## Materials and methods

### Patient population

A single-institution retrospective case series informed by a prospectively maintained database of patients with SNUC was performed. The study was approved by the University of Michigan Institutional Review Board (HUM00080561). Patients with a history of sinonasal undifferentiated carcinoma treated at the University of Michigan were included in the clinical dataset (*n* = 46). Pathology descriptions for the cohort are listed in Supplemental Table 1a. Inclusion criteria for genomic, copy number and transcriptome analysis is as follows: 1) Patients with sinonasal undifferentiated carcinoma as confirmed by our board-certified pathologist (J.B.M.); 2) Blocks maintained in the University of Michigan pathology archive; 3) Sufficient DNA or RNA yield for next generation sequencing. Additionally, a prospective patient was consented to our University of Michigan IRB-approved MiOTOseq precision medicine program (HUM00085888) as described [20]. In total, there were 5 patients who met inclusion criteria for analysis, and demographics are shown in Supplemental Table 1b.

### Survival analysis statistics

Survival was calculated using Kaplan-Meier analysis and outcomes were compared using Log-rank analysis. Multivariate cox regression analysis was performed using Backward Wald method with an inclusion of variables with *p*-values < 0.1. Statistical analysis was performed using SPSS v26 (IBM, Armonk, NY). Kaplan-Meier curves were created using Prism v8 (Graphpad, San Diego, CA).

### Cell line

The patient derived SNUC cell line, MDA8788–6, was generously provided by MD Anderson. Generation of this cell line was previously described by Takahashi *et.al* [21]. Cells were cultured in a humidified incubator at 37 °C with 5% (vol/vol) CO2 in DMEM with 10% FBS, 1X Pen/Strep, 1X NEAA. Cells were genotyped to confirm the STR profile of the cell line (Supplemental Table 2) as previously described [22].

### DNA isolation

DNA was isolated from formalin fixed, paraffin-embedded (FFPE) samples following the manufacturer’s protocol for AllPrep DNA/RNA FFPE kit (Qiagen, Hilden, Germany) as previously described [23, 24]. Tumor and adjacent normal regions were identified on H&E stained slides and aligned to tissue paraffin blocks. An 18-gauge sterile needle was used to core 2–4 samples from each region. Deparaffinization was performed using the xylene/ethanol method with the only modification being that samples were digested using proteinase K at 56 °C for 20–24 min. DNA isolation was then completed using the Allprep Isolation kit (Qiagen, Hilden, DE) following manufacturer protocol. Each sample was analyzed using a Nanodrop spectrophotometer for purity (260:230 and 260:280 ratios) and concentration was determined using 1uL of sample with the Qubit 2.0 Fluorometer and measured with a bioanalyzer as described [25]. DNA extraction for MDA8788–6 cell line was performed using Wizard® Genomic DNA Purification Kit (Promega, Madison, WI).

### DNA sequencing

Genomic DNA from each tumor and adjacent normal specimen was submitted for sequencing to the University of Michigan’s DNA sequencing core for exome sequencing using both the DNA TruSeq Exome Library Preparation kit (Illumina, Catalogue number FC-150-100x; SNUC2, SNUC5, SNUC8, SNUC10) and the Roche NimbleGen V3 capture kit (SNUC1). DNA from the MDA 8788–6 cell line was sequenced as described [26]. Libraries were prepared according to the manufacturer’s instructions. Libraries were then paired end sequenced to 125 nucleotides as part of pool with an average of 4 samples per lane on an Illumina HiSEQ4000 yielding an average depth of greater than 90x per sample.

### Exome variant calling

Quality of the sequencing reads was assessed using FastQC v.0.11.5. Because the reads had adapter contamination as well as a high k-mer content at the start of the reads, trim galore v0.4.4 was used to remove adapters and trim reads. Reads were aligned to the hg19 reference genome using BWA v0.7.1. Mapping was followed by marking duplicates using PicardTools v1.79. Base quality score recalibration was done using GATK v3.6 and this was the last step in preparing the reads for variant calling. Samtools v1.2 was used to create pileup files for each tumor-normal pair. Varscan v2.4.1 was used to call variants from these mpileup files using the somatic mode of the variant caller. Goldex Helix Varseq v1.4.6 was used to annotate variants. All variants in the introns and intergenic regions were filtered out. Variants with more than 5 reads supporting the alternate allele in the tumor samples were considered as true positives.

### Copy number analysis

Aberration Detection in Tumor Exome (ADTEx) v.2.0 was used to make copy number estimation calls from the pre-processed tumor-normal BAM files which were also used for variant calling. A state from 0 to 4 was assigned by the software based on its estimated copy number. State 0 corresponds to a homozygous deletion, 1 corresponds to a heterozygous deletion. A normal copy number is denoted by state 2. States 3 and 4 represent a gain and amplification respectively.

### Microsatellite instability (MSI) detection

MSIsensor was used to detect somatic MSI loci from the tumor-normal sample pairs as described previously [27]. The software assigns a status to each sample pair based on an instability score calculated based on a threshold of more or less than 3.5% of called microsatellites having alterations. We present this score as well as the overall percentage of microsatellite alterations for each tumor-normal pair.

### Sanger sequencing

Excess DNA from above was used to validate mutation calls in novel genes. Primers were designed using MITprimer3 to amplify a small region surrounding the nominated single nucleotide variants (SNVs) as described in Supplemental Table 3. Polymerase chain reactions (PCRs) were optimized for each primer pair on cell line genomic DNA and then used to amplify the regions from tumor and adjacent normal DNA using Platinum Taq DNA High Fidelity polymerase (ThermoFisher, Waltham, MA). PCRs products were then visualized on an eGel (Invitrogen, Waltham, MA) and purified using the a PCR purification kit (Qiagen, Valenica, CA) as described [28] and submitted for Sanger sequencing at the University of Michigan’s DNA sequencing core. Results were visualized using the LaserGene software suite.

### Cell line RNA sequencing and analysis

Total RNA from the MDA8788–6 sample underwent standard QC and was submitted for RNA sequencing to the University’s DNA sequencing core as previously described [26, 29]. Briefly, the Illumina Stranded RNAseq kit was used and libraries were sequenced on an Illumina HiSEQ4000 using 75 nt paired end approach. Quality of the RNA sequencing reads was determined using FastQC v0.11.5 and we did not identify any quality issues. We then used a two-step alignment protocol of Star v2.5.3a to map the reads and genome index files were first generated using the reference human genome and annotated transcriptome files. In the second step, we then used the index files to guide read mapping. Samtools v1.9 and Picard v2.4.1 were used to retain only uniquely mapped reads and FPKM was computed using Cufflinks v2.2.1 with default parameters, with the exception of modifying “--max-bundle-frags” to 100,000,000. This modification was made to avoid raising of the HIDATA flag at loci that have more fragments than the pre-set threshold for every locus.

### Fusion gene analysis

FusionCatcher (v1.00) is a software package designed to look for gene fusions, translocations and rearrangement events using paired end RNA-Seq data and was used to identify novel gene fusions in the MDA8788–6 cell line.

### Linked read sequencing

High molecular weight DNA was isolated from the SNUC cell line by lysing 1.5 million cells overnight at 37° with lysis buffer (10 mM Tris-HCl, 400 mM NaCl, 2 mM EDTA), 10% SDS, and a proteinase K solution (1 mg/mL Proteinase K, 1% SDS, 2 mM EDTA). Following overnight lysis, DNA was salted out of the solution with 5 M NaCl for 1 h at 4° and precipitated with ice cold ethanol for 5 h at − 20 °C. High molecular weight DNA was eluted in TE buffer; the quality and integrity of the DNA was assessed using the Tapestation Genomic DNA ScreenTape kit (Agilent). The DNA was submitted to the University’s DNA sequencing core for 10x based linked read library generation and sequencing on an Illumina NovaSeq6000 with 300 nt paired end run. Samples were de-multiplexed and FastQ files with matched index files were generated using Long Ranger Version 2.2.2. Data was visualized using Loupe software package, Version2.1.1 (2.4).

### Fusion gene knockdown

All siRNA including ON-TARGETplus Non-targeting Control siRNA, ON-TARGETplus GAPDH Control siRNA, and a custom siRNA targeting the PGAP3-SRPK1 fusion site were purchased through Dharmacon (Lafayette, CO). Each siRNA was reconstituted at a concentration of 1 nmol/50 uL in 1X siRNA buffer (DHarmacon, Lafayette, CO). MDA8788–6 cells were plated at a concentration of 250,000 cell per well in 3 mL growth media. The following day all media was removed and cells were starved in 1 mL of serum DMEM for 3 h. Each siRNA was prepared by adding 400uL of OPTI-MEM (Gibco, Waltham, Massachusetts) with 24uL of siRNA and left to equilibrate for 5 min. Separately, 24uL of oligofectamine (Invitrogen, Carlsbad, CA) was added to 96uL of OPTI-MEM. After 5 minutes the two mixtures are added together and allowed to equilibrate at room temperature for 20 min. Cell were then treated with 250uL of siRNA mixture containing buffer only, Non-targeting siRNA, PGAP3-SRPK1 fusion siRNA, or GAPDH siRNA. After 3 h 2.5 mL of growth medium was added to each well. The following day cells were harvested in 700uL of QIAzol Lysis Reagent (Qiagen, Valencia, CA) and proceeded directly to RNA extraction or stored at minus 80 °C for future extraction. RNA extraction was performed using RNeasy Mini Kit (Qiagen, Valenica, CA) per manufacturer’s instructions. RNA sequencing of the fusion knockdown was also performed as above. Briefly, extracted total RNA from MDA8788–6 NT siRNA and PGAP3-SRPK1 fusion siRNA were submitted to the University’s DNA sequencing core and processed as above (Illumina Stranded RNAseq kit was used and libraries were sequenced on an Illumina NovaSEQ6000 using 300 cycle paired end approach).

### Quantitative polymerase chain reaction (qPCR)

Confirmation of successful siRNA knockdown and validation of RNAseq findings were performed with qPCR. Following RNA extraction, cDNA synthesis was performed using SuperScript™ III First-Strand Synthesis System (Invitrogen, Carlsbad, CA) and qPCR was performed using QuantiTect SYBR Green PCR Kit (Qiagen, Valencia, CA) and run on QuantStudio5 (Applied Biosystems, Foster City CA). Targets included *SRPK1*, *PGAP3*-*SPRK1* fusion, *GAPDH*, *HSDL2*, *CCND1*, *FOXO4*, *Beta-Actin*, *HRPT*, and *RPL-19*; primer sequences are listed in Supplemental Table 4. Analysis was performed using the ^2ΔΔ-Ct^ method [30].

## Results

### Survival analysis

**Fig. 1:**
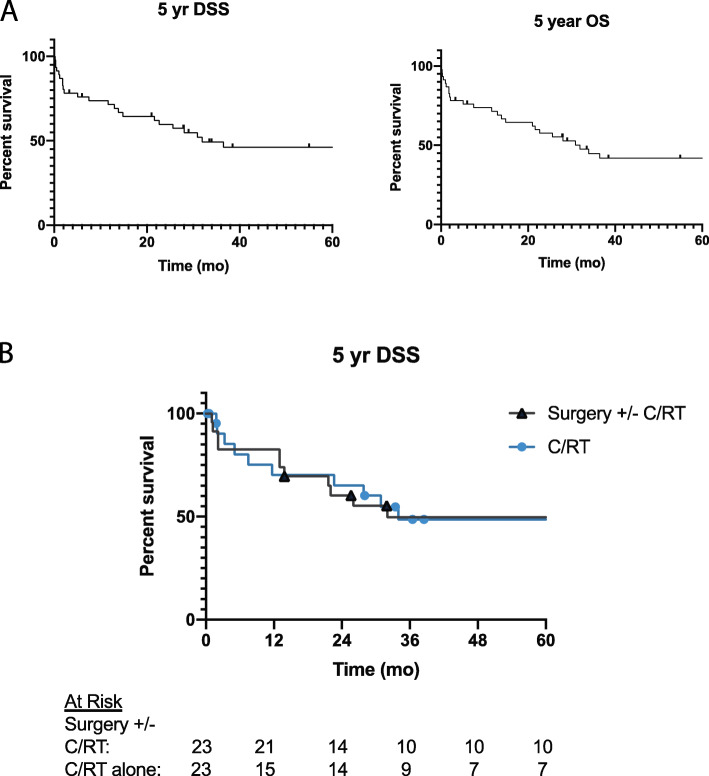
Kaplan-Meier Survival Curves. **A**) Five-year disease specific survival (DSS) and overall survival (OS) for SNUC cohort. **B**) Five-year DSS stratifying by the use of surgery with or without chemotherapy and radiation (CRT) compared to CRT alone

Forty-six patients were included in the survival analysis. The median age at diagnosis 53 years with a range from 19 to 87 years. Median follow up time was 28 months (range <  1 month – 23 years). Two patients (4.3%) were treated with surgery alone, 21 patients (44%) with surgery in addition to adjuvant radiation, chemotherapy, or both (CRT) and 23 patients (49%) with CRT alone. Twenty-five patients (53%) had persistent or recurrent disease after treatment. The median time to recurrence was 2.8 months. Of these 64% were locoregional recurrence or persistence while 32% were distant failures. Five-year overall survival was 42% (95% CI 27–56%) and 5-year disease specific survival was 46% (95% CI 30–61%) as seen in Fig. 1a. There was no significant difference in 5-year DSS when stratifying by use of surgery +/− CRT and CRT alone (50% [95% CI 27–69%] vs. 49% [95% CI 25–68%], *p* = 0.85), Fig. 1b. Multivariate analysis was performed using backward Wald cox regression. Variables included in the model were T-stage, nodal disease, tobacco use and treatment type (surgery +−/ CRT vs. CRT alone). Only tobacco use was found to be significantly associated with decreased survival (HR 5.1 [95% CI 1.7–15], *p* = 0.004).

### Exome sequencing of SNUC tumors

We were able to isolate high quality DNA that met our quality control standards for sequencing from 5 retrospective SNUC and matched normal samples that were advanced for full exome sequencing. Within our 5 sample cohort, we had 2 patients that died within 2 years of diagnosis and 3 patients that survived for more than 5 years after diagnosis (Table 1). Using these tumor and adjacent normal DNA samples, we sequenced exomes to an average depth of 256,352,493 yielding an average of 218,566,135 uniquely mapped reads in each sample, for an average coverage of >100X per tumor (Supplemental Table 5).

**Table 1:** Patient and tumor characteristics

Sample ID	Tobacco Use (Current, Former, Never)	Site	TNM Classification	Initial Treatment	Died of Disease (Y/N)	Survival Months	Histology
SNUC1	Never	Nasal Cavity	T4bN0M0	Surgery, Adjuvant RT	N	144	
SNUC2	Former	Maxillary Sinus	T3N0M0	CRT	Y	< 1	
SNUC5	Never	Nasal Cavity	T3N0M0	Surgery, Adjuvant CRT	N	125	
SNUC8	Never	Ethmoid Sinus	T4bN0M0	Surgery, Adjuvant CRT	N	130	
SNUC10	Never	Nasal Cavity	T4bN0M0	Surgery, Adjuvant CRT	Y	3	

**Fig. 2:**
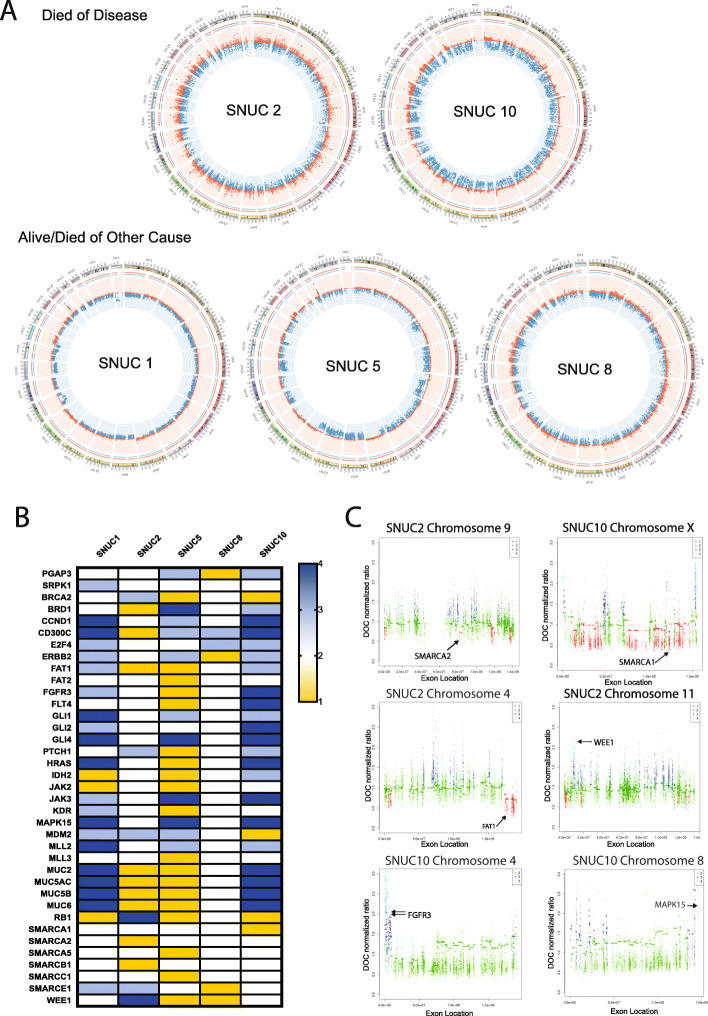
Copy Number Variation (CNV) Analysis. **A**) Circos Plots depicting copy number variation for each sample. For patients who died of disease, 2/2 had increased copy number variations, compared to only 1/3 in the group of patients who did not die of disease. **B**) Heatmap depicting copy number for key genes; 1- copy loss, 2 –copy neutral, 3- copy gain, and 4 – amplification. **C**) Manhattan plots for highlighted genes with focal copy alterations

Using this data, we first generated copy number calls for broad genomic regions and assessed the global view of the copy number variation in Circos plots (Fig. 2a, Supplemental Tables 6A-E). These plots demonstrated that 3/5 tumors showed a much higher level of copy number variation as compared to the other samples with frequent high level amplifications in chromosomes 4, 17, 19 and 22. Genes with copy gain or amplifications in multiple tumors included: *CD300C* which plays a role in innate immunity and antigen presentation via MHC class I and is a negative regulator of CD4 and CD8 T cells [31], *JAK3*, *E2F4* and *GLI1,* which have canonical and non-cannonical roles in tumor cell proliferation respectively [32, 33], *MDM2* which targets p53 for degradation [34], and *MLL2* which may play a role in epithelial-mesenchymal transition (EMT) [35]. We also identified copy gains in *ERBB2* in 3/5 samples (Fig. 2b) suggesting a potential role of *ERBB2* function in a subset of SNUCs, which has been previously noted [18]. Moreover, we also identified copy number losses in *SMARC* family genes (*SMARCA1, SMARCA2, SMARCA5, SMARCB1, SMARCC1 and SMARCE1*) in numerous samples consistent with previous reports of the recurrence of alterations to this gene family in SNUC [16]. Focal amplifications were identified in *WEE1*, *FGFR3* and *MAPK15* while focal copy losses were identified in *FAT1* and *SMARCA2* (Fig. 2c).

**Fig. 3:**
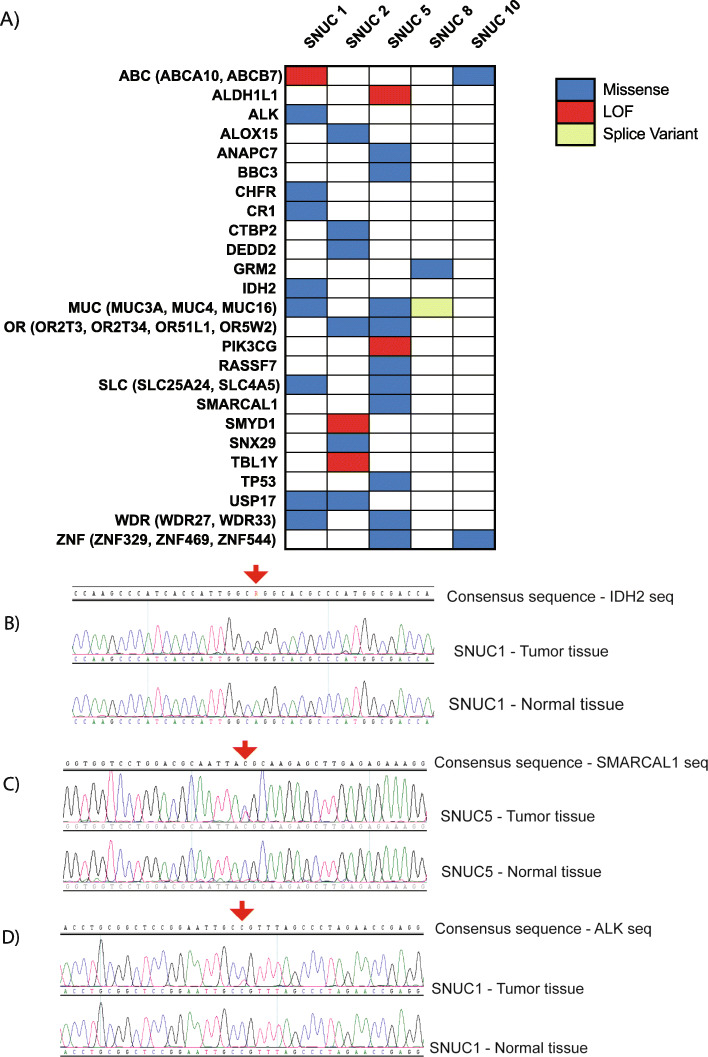
**A**) Oncoplot depicting high confidence non-synonymous somatic alterations in key genes in five SNUC samples. Sanger Sequencing Validation of Single Nucleotide Variations. **B**) *IDH2* p.Arg172Gly in SNUC1. **C**) *SMARCAL1* p.Thr742Met in SNUC5. **D**) *ALK* p.Gly872Ser

Analysis of SNV data revealed an average of 23.6 (range of 5–63) high confidence non-synonymous somatic alterations per tumor (Supplemental Figure 1). Oncoplots for recurrently altered as well as targetable or cancer-relevant genes are shown in Fig. 3a and an extended list of the mutations with functional annotations and pathogenicity scores is shown in (Supplementary Tables 7, 8, 9). From this analysis, we identified a mutation in *IDH2* (p.Arg172Gly), which is a gene previously associated with SNUC [15, 17], as well as new mutation disrupting *SMARCAL1* (p.Thr742Met; Fig. 3b, c) suggesting alternative pathways may be implicated in tumorigenesis in these samples. Further, we identified potentially targetable alterations including an *ALK* p.Gly872Ser (Fig. 3d). We also identified two different ABC transporter genes, *ABCA10* (p.Glu507Ter) and *ABCB7* (p.Glu342Gln), suggesting a general pathway for drug resistance that may explain why some tumors respond poorly to chemotherapy.

Finally, we characterized microsatellite instability in each of the tumors using the MSIsensor software package, which assigns an instability score to each tumor/normal pair. These data revealed an average of 16.4 somatic sites and a median % of 4.99 (range 0.72–6.95). Two of the SNUC samples were found to be microsatellite stable while three were found to be unstable (Supplemental Table 10).

### Exome and RNAseq analysis of SNUC cell line (MDA8788–6)

Our colleagues recently derived the first SNUC cell line, MDA8788–6, and we performed exome sequencing on this cell line model with subsequent SNV annotation using our previously established informatics pipelines for head and neck cell lines without available matched normal DNA. This sequencing yielded 225,772,526 reads, of which 99.7% uniquely mapped to the genome yielding an average coverage of >100X (Supplemental Table 11). As no matched normal was available, joint calling was completed with our UM-SCC cell lines as described [26, 29], and filtering to retain only heterozygous calls and remove any SNV previously reported in dbSNP, yielded 563 potential single nucleotide variants, of which 182 were categorized as missense, frameshift or stopgains (Supplemental Table 12). Similar filtering for insertion/deletion calls yielded 779 potential INDELs, of which 39 were categorized as frameshift INDELs (Supplemental Table 13). Among these alterations, we identified *KMT2B* S421P and *NOTCH1* T2483 missense mutations, which may have an important role in pathogenesis of this tumor model given the previously established role of these genes in tumorigenesis.

**Fig. 4:**
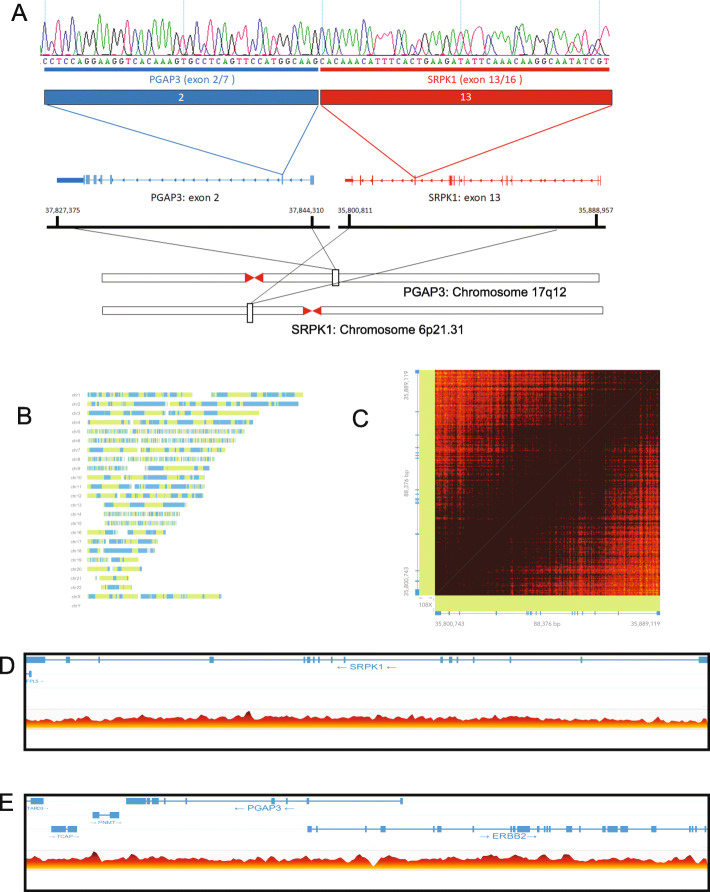
*PGAP3-SRPK1* fusion gene and linked read genome sequencing. **A**) Schematic representation of the novel *PGAP4-SRPK1* fusion gene. Exon 2 of *PGAP3* is fused to exon 13 of *SPRK1*. **B**) Phase map summarizing linked read sequencing data as reported by the LongRanger and Loupe Analysis pipeline. **C**) Linked read matrix map showing high density coverage of linked reads over the SRPK1 locus. Dark brown indicates >30x coverage. **D**, **E**) Linear view of linked read data highlighting structural variations (indicated with colored lines, with horizontal bars indicating the direction of each paired read), for SRPK1 (**D**) and PGAP3 (**E**). Read coverage is shown on the bottom, ranging from white/yellow = zero reads to dark red = 26 reads

Next, we performed paired end RNA sequencing to discover potential gene fusions in the cell line using the FusionCatcher algorithm. This analysis called 107 potential gene fusions in the cell line to a variety of known oncogenes and previously described fusion genes from other cancers (Supplemental Table 14). We focused validation studies on inframe gene fusions, with at least 10 supporting reads that spanned the fusion junction. Importantly, we were able to validate the presence of a novel fusion between *PGAP3* exon 2 and *SRPK1* exon 13 (Fig. 4a). The resulting fusion gene creates an in-frame fusion gene predicted to encode a 244 amino acid, with predicted isoelectric point (pI) of 5.87 and molecular weight of 27.6 kDa protein (Supplemental Figure 2) [36, 37]. The resulting fusion protein is predicted to retain the SRPK1 protein kinase domain, which normally regulates constitutive and alternative pre-mRNA splicing machinery through phosphorylation of SR proteins [38].

To then test the hypothesis that large scale structural rearrangements drove fusion formation, we performed 10X linked read genome sequencing on high molecular weight DNA isolated from the MDA8788–6 cell line. We obtained 848,842,686 total reads and averaged 36.9x coverage, with 96.1% of the DNA molecules of > 20 kb in length and 58% greater than 100 kb (Supplemental Table 15). Long ranger pipeline analysis phased 99.2% of SNPs resulting in a maximum phase block of 47.9 MB (Fig. 4b). The analysis identified 888 large structural variant calls in the genome. Contrary to our hypothesis, there were no structural rearrangements around *PGAP3* or *SRPK1* identified (Fig. 4c-e) suggesting that the fusion is formed at the RNA level, possibly by trans-splicing events. However, we did identify structural alterations involving *ZNF546* and *AXL* (Chr19:40,490,000 and Chr19:41,760,000, 1.27 MB duplication, quality score 1000) as well as *CREB3L2* and *BRAF* (Chr7:137,630,000 and Chr7:140,490,000, 2.86 MB duplication) suggesting a potential role for these oncogenes in pathogenesis of this SNUC model (Supplemental Figure 3).

**Fig. 5:**
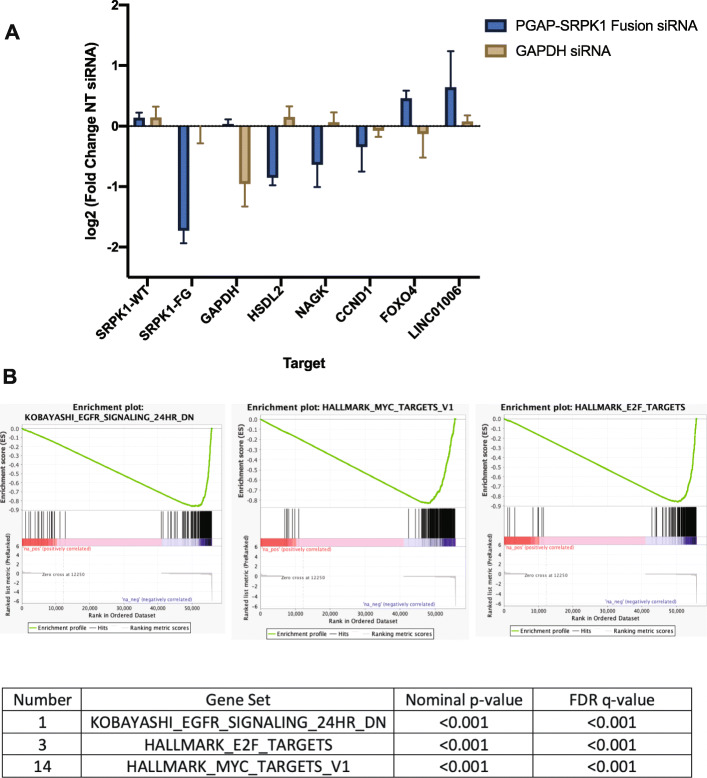
RNAseq and gene set enrichment analysis (GHSEA) results for PGAP3-SRPK1 fusion knockdown. **A**) qPCR of MD8788–6 treated with PGAP3-SRPK1 siRNA compared to NT siRNA. Results are quantified as log2 fold change of NT siRNA samples. Results demonstrate knockdown of PGAP3/SRPK1 mRNA without changes in WT SRPK1 expression and confirm RNAseq results showing decreases in HSDL2 and CCDN1 and a small increase in FOXO4 with PGAP3-SRPK1 knockdown (*n* = 2 replicates). **B**) GSEA results demonstrating a negative correlation with KOBAYASHI_EGFR_SIGNALING_24HR_DN, HALLMARK_E2F_TARGETS, and HALLMARK_MYC_TARGETS_V1 pathways in samples with PGAP3-SRPK1 knockdown

Finally, to test for potential functional roles of the *PGAP3*-*SRPK1* fusion, we performed siRNA-mediated knockdown of the fusion transcript and submitted RNA for complete transcriptome sequencing. This data demonstrated that knockdown of *PGAP3-SRPK1* fusion results in significantly decreased *HSDL2, NAGK*, and *CCND1* RNA expression and a modest increase in *LINC01006* and *FOXO4* suggesting *PGAP3-SRPK-1* may play a role in regulation of these genes (Fig. 5a). In fact, we identified 122 genes that were > 2-fold upregulated and 112 genes > 2-fold downregulated in the knockdown relative to control. Further, gene set enrichment analysis of the differentially expressed rank list found enrichment of KOBAYASHI_EGFR_SIGNALING_24HR_DN, HALLMARK_E2F_TARGETS, and HALLMARK_MYC_TARGETS_V1 pathways (Fig. 5b, Supplemental Table 16). Collectively, this RNA sequencing data analysis suggests that the gene fusion has a pivotal role in the hallmark signaling pathways.

## Discussion

Here we report survival outcomes for a cohort of 46 SNUC patients treated with CRT or surgery +/− CRT. Survival analysis from our cohort is congruent with previous reports of low survival rates [1, 4–9] and shows little differences in survival when stratifying by treatment modality. Multivariate analysis revealed that only tobacco use was an independent predictor of poor outcomes in our cohort. The lack of robust clinical predictors highlights the need for more in-depth understanding of molecular markers that may predict treatment outcomes.

In this study, we confirm the presence of previously noted alterations in *IDH2*, *SMARC* family members, and *ERBB2* from initial targeted sequencing studies. Previous studies have noted high rates of *IDH2* mutations ranging from 55 to 84% [15, 17, 39] and have identified R172X as a hotspot location. While only 1/5 of our samples contained an *IDH* mutation, this did occur at the R172 codon. Similarly, prior studies have cited loss of *SMARCB1* in 33–43% of SNUC samples and have demonstrated worse outcomes in these patients [40, 41]. Notably, while we did not find a high frequency of *SMARCB1* mutations, we did identified copy number alterations in *SMARCB1 in addition* to other SMARC family members. These data suggest that deregulation of the *SWI/SNF* nucleosome remodeling complex (consisting of known tumor suppressors *SMARCB1*, *SMARCA4*, *PBRM1*, *ARID1B*, and *ARID2*), through one of its many components, is a critical step in disease progression in SNUCs [42]. *SMARCB1* has been implicated in numerous other solid cancers as a tumor suppressor gene, including sarcomas, carcinomas and rhabdoid tumors of varying sites [43]. It appears to have tumor suppressor functions in inhibiting cell cycle and proliferation via the p16-Rb-E2F and Wnt/Beta-catenin pathways, among others [43]. *SMARCA2* function and expression may also play a critical role in response to specific targeted therapies (particularly with EZH2 inhibition) in tumors with *SWI/SNF* dysregulation [44], suggesting a potential role for EZH2 inhibitors in SNUCs. The remainder of our samples however lacked the traditional *SMARCB1* or *IDH* mutations implying diversity in SNUC tumorigenesis and suggesting the importance of identifying novel alterations within these SNUC tumors.

Previous isolated reports of SNUCs have identified overexpression or amplification of growth factor receptors [18, 19, 45] and in this study, we have identified genetic alterations in *ERBB2* and *FGFR* family growth factor receptor genes as well as *ALK*, suggesting potential targetable option in SNUCs. In a previous study of a SNUC cell line, high *ERBB2* expression was identified with a notable response to ERBB2 inhibition [18]. *FGFR3* alterations have been implicated in head and neck cancer and in vitro and in vivo studies suggest a promising role for FGF inhibition in head and neck tumors [46–48]. Further, a recent publication by Takahashi et al. identified a 34 gene signature differentiating responders from non-responders after induction chemotherapy [49]. Critical pathways highlighted in this work included *PI3K* and *JAK/STAT*. Our work similarly identified alteration within *PIK3CG* as well as recurrent alterations within the *JAK/STAT* pathway. Given the diverse, but potentially actionable set of alterations that our data defined, these results suggest a role for in depth molecular analysis of this rare disease in order to gain insight into molecular alterations that may drive discovery of future therapeutics, and potentially guide individual patient treatment options.

Finally, this study identifies a novel fusion of *PGAP3-SRPK. SRPK1* has been previously characterized to drive cell proliferation, migration, and invasion in colorectal and gastric cancers [50–53] suggesting that the fusion protein may have oncogenic consequences in the SNUC cell line. CNV analysis additionally revealed copy gain in one tumor in the *SPRK1* gene. Knockdown of the PGAP3-SRPK1 fusion gene resulted in changes in expression of *CCND1*, *FOXO4* and most significantly a decrease in *HSDL2* and *NAGK* suggesting a functional role for this novel fusion gene. Unfortunately, insufficient RNA prevented evaluation of SNUC tissues for presence of the fusion. This is the first study to date to suggest a role of *SRPK-1* in sinonasal undifferentiated carcinoma.

Limitations in interpretation of the novel *PGAP3-SRPK1* gene fusion do exist. For example these results may represent a trans-splicing event such as that described by Li et al. In this paper, the authors demonstrate the presence of chimeric JAZF1-JJAZ RNA in normal endometrial tissue lacking the *JAZF1-JJAZ* fusion [54]. Given we have not yet performed protein validation of the PGAP-SRPK-1 fusion it is possible that this represents chimeric RNA that occurred in a trans-splicing event. Further it is also possible that a DNA rearrangement was missed by our sequencing. We had on average > 20 reads covering the *PGAP3* and *SRPK1* genes (read coverage of each gene is depicted in Fig. 4d and e), with a slight gap in read coverage between exon 1 and 2 of *PGAP3* that corresponds to the potential breakpoint in that gene, so it is possible that the linked read analysis missed the DNA breakpoint because of low sequence ability, or other library specific issues.

## Conclusion

Given the rarity of this tumor, it will be challenging to characterize a large cohort of patients. Nevertheless, we believe this initial analysis of five SNUCs represents a valuable preliminary guideline of the mutational landscape of SNUCs and identifies multiple recurring mutations and pathway alterations. These may be of particular interest both as prognostic biomarkers in larger cohort studies, and as potentially targetable therapeutic options. Consequently, the alterations identified here represent promising targets for future SNUC studies and support a potential pathogenic role in other cancers. Due to both the infrequency and highly aggressive nature SNUC, our hope is this study will serve as primer to advance therapeutic concepts developed for other malignancies against these pathways into future SNUC therapies.

## Supplementary Information

**Additional file 1: Supplemental Figure 1.** High confidence somatic mutations and INDELS is depicted for each sample.

**Additional file 2: Supplemental Figure 2.** Predicted protein structure of *PGAP3-SRPK1* fusion gene including predicted active site.

**Additional file 3: Supplemental Figure 3.** Linked read genome sequencing identifies structural variations in the MDA8788-6 genome involving ZNF546 and AXL as well as CREB3L2 and BRAF.

**Additional file 4: Supplemental Table 1**a and b.

**Additional file 5: Supplemental Table 2.** SNUC STR Profile.

**Additional file 6: Supplemental Table 3.** Sanger Sequencing Primers.

**Additional file 7: Supplemental Table 4.** Primer Sequences.

**Additional file 8: Supplemental Table 5.** Whole Exome Sequencing QC.

**Additional file 9: Supplemental Table 6A.** Copy Number Variation Data (SNUC 1). **Supplemental Table 6B.** Copy Number Variation Data (SNUC 2). **Supplemental Table 6C.** Copy Number Variation Data (SNUC 5). **Supplemental Table 6D.** Copy Number Variation Data (SNUC 8). **Supplemental Table 6E.** Copy Number Variation Data (SNUC 10).

**Additional file 10: Supplemental Table 7.** Single Nucleotide Variants (SNV) Analysis - SNUC samples.

**Additional file 11: Supplemental Table 8.** INDELs Annotation - SNUC samples.

**Additional file 12: Supplemental Table 9.** VEST.

**Additional file 13: Supplemental Table 10.** Microsatellite Status.

**Additional file 14: Supplemental Table 11.** Cell Line Exome QC.

**Additional file 15: Supplemental Table 12.** Single Nucleotide Variant (SNV) Annotations.

**Additional file 16: Supplemental Table 13.** INDELs Annotations.

**Additional file 17: Supplemental Table 14.** Gene Fusion Data.

**Additional file 18: Supplemental Table 15.** Long Ranger Data.

**Additional file 19: Supplemental Table 16.** Knockdown Gene Set Enrichment Analysis Summary.

## Data Availability

All data generated or analyzed during this study are included in this published article and its supplementary information files as well as deposited online (SRA PRJNA668767).
