# Supplementary material for: Genetic analysis of sinonasal undifferentiated carcinoma discovers recurrent SWI/SNF alterations and a novel PGAP3-SRPK1 fusion gene
Source: BMC Cancer. 2021 May 29;21:636. doi: 10.1186/s12885-021-08370-x (PMC8164750; doi:10.1186/s12885-021-08370-x)
Supplement: Supplementary file 1 — Additional file 1: Supplemental Figure 1. High confidence somatic mutations and INDELS is depicted for each sample. [file 12885_2021_8370_MOESM1_ESM.docx]

**Supplemental Figure 1:** High confidence somatic mutations and INDELS is depicted for each sample.
