# Supplementary material for: Genetic analysis of sinonasal undifferentiated carcinoma discovers recurrent SWI/SNF alterations and a novel PGAP3-SRPK1 fusion gene
Source: BMC Cancer. 2021 May 29;21:636. doi: 10.1186/s12885-021-08370-x (PMC8164750; doi:10.1186/s12885-021-08370-x)
Supplement: Supplementary file 2 — Additional file 2: Supplemental Figure 2. Predicted protein structure of PGAP3-SRPK1 fusion gene including predicted active site. [file 12885_2021_8370_MOESM2_ESM.pdf]

Supplemental Figure 2: Predicted protein structure of PGAP3-SRPK1 fusion gene including predicted active site (Ref 37,38).

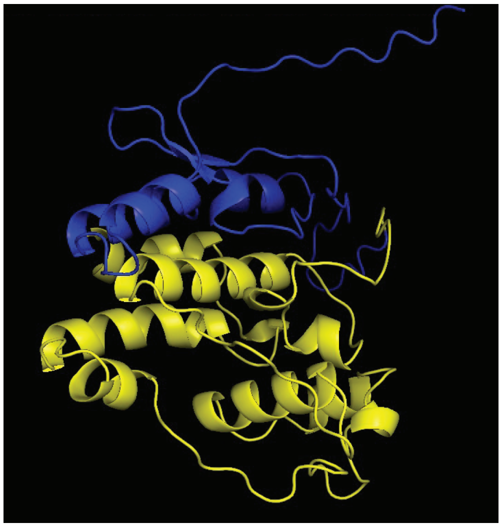

PGAP
SRPK-1

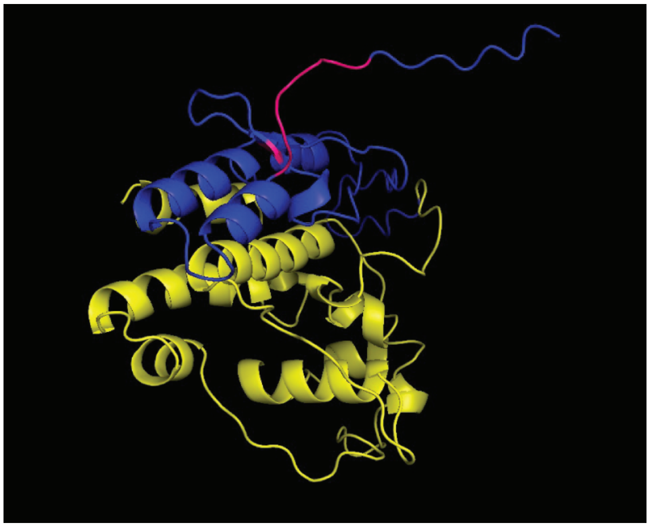

PGAP
SRPK-1
Predicted Binding Site

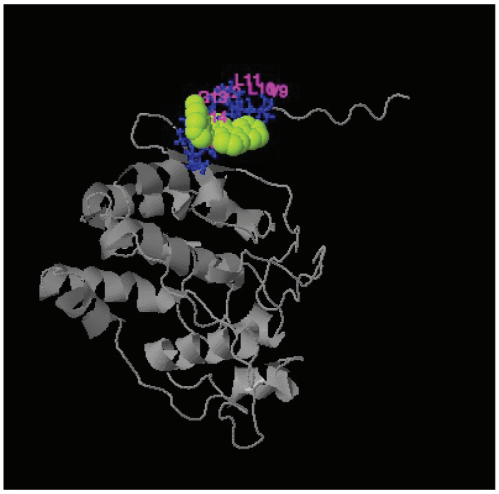

Predicted Binding Pattern

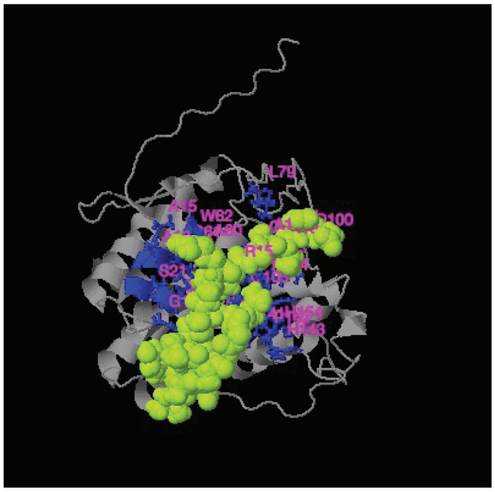

Alternative Predicted Binding Site

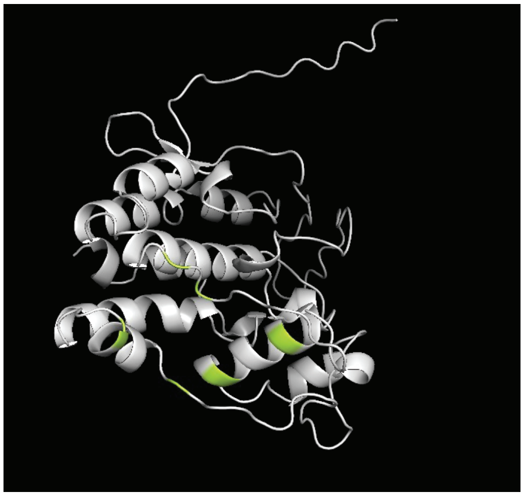

SRPK-1 Active Binding Site
