# Supplementary material for: Genetic analysis of sinonasal undifferentiated carcinoma discovers recurrent SWI/SNF alterations and a novel PGAP3-SRPK1 fusion gene
Source: BMC Cancer. 2021 May 29;21:636. doi: 10.1186/s12885-021-08370-x (PMC8164750; doi:10.1186/s12885-021-08370-x)
Supplement: Supplementary file 3 — Additional file 3: Supplemental Figure 3. Linked read genome sequencing identifies structural variations in the MDA8788-6 genome involving ZNF546 and AXL as well as CREB3L2 and BRAF. [file 12885_2021_8370_MOESM3_ESM.pdf]

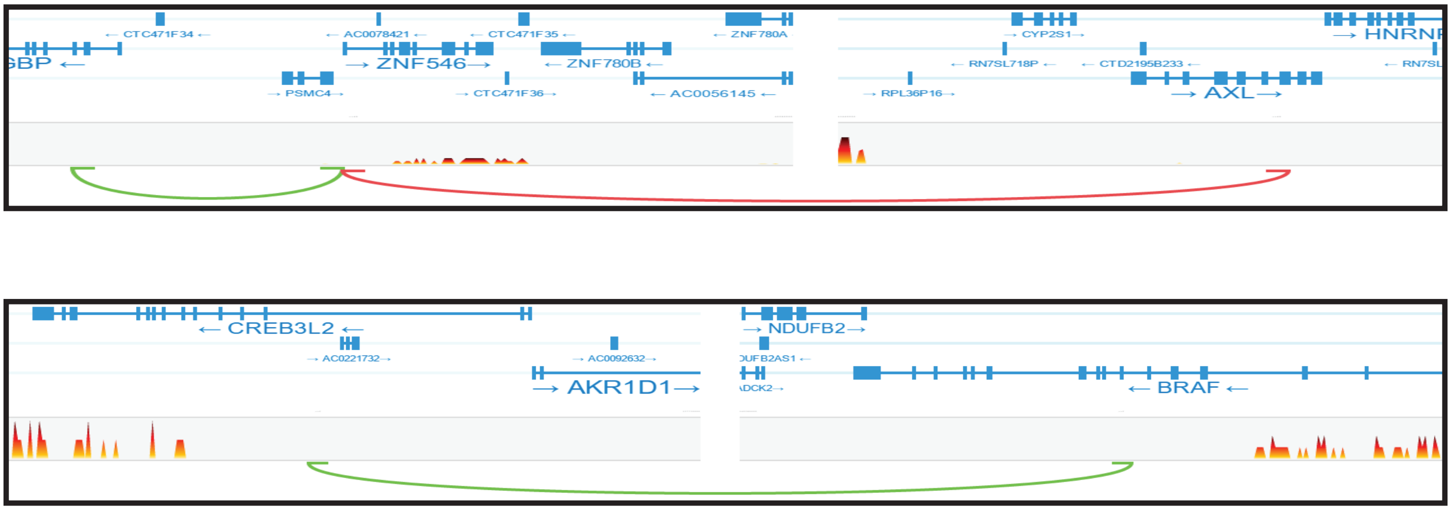

Supplemental Figure 3: Linked read genome sequencing identifies structural variations in the MDA8788-6 genome involving ZNF546 and AXL as well as CREB3L2 and BRAF.
