# Supplementary material for: Genetic analysis of sinonasal undifferentiated carcinoma discovers recurrent SWI/SNF alterations and a novel PGAP3-SRPK1 fusion gene
Source: BMC Cancer. 2021 May 29;21:636. doi: 10.1186/s12885-021-08370-x (PMC8164750; doi:10.1186/s12885-021-08370-x)
Supplement: Supplementary file 4 — Additional file 4: Supplemental Table 1a and b. [file 12885_2021_8370_MOESM4_ESM.docx]

Supplemental Table 1a

| **Initial Tumor Date** | **Pathology Details** |
| --- | --- |
| 12/21/06 | The tumor cells are diffusely positive for cytokeratin, focally positive for CD56 and negative for synaptophysin and chromogranin. These findings rule out a diagnosis of a neuroendocrine tumor and support the diagnosis of sinonasal undifferentiated carcinoma |
| 2/14/07 | The following special stains were performed: S-100, synaptophysin, CAM 5.2, cytokeratin AE1/AE3, melan-A, CD45, CD3, CK 5/6, p63 and CD20. The tumor cells are focally positive for CAM 5.2 and AE1/AE3. The tumor cells are negative for CD3, CD20, CD45, melan-A, S-100, P63, and cytokeratin 5/6. These findings, in conjuntion with the hitologic features of the cell population, are most compatible with sinonasal undifferentiated carcinoma. |
| 10/1/04 | Right nasal cavity, biopsies (S04-41706, 11/04/04): Poorly-differentiated carcinoma, consistent with sinonasal undifferentiated carcinoma. Immunohistochemical stains are positive for CAM5.2 and cytokeratin AE1/3. Stains are negative for chromogranin, synaptophysin, NSE and cytokeratin 34 beta E12. |
| 2/8/07 | Posterior right nasal cavity, biopsy: Poorly differentiated carcinoma, most in keeping with sinonasal undifferentiated carcinoma. The tumor cells are positive for cytokeratin CAM5.2 and negative for high molecular weight cytokeratin 34BE12, cytokeratin AE1/AE3, chromogranin, synaptophysin, CD56, and S-100. In situ hybridization for EBER is negative. |
| 9/12/03 | Left maxillary sinus mass, partial excision: Sinonasal undifferentiated carcinoma (SNUC). |
| 11/23/16 | Right nasal mass, biopsies: High-grade carcinoma consistent with sinonasal undifferentiated carcinoma. See comment.     COMMENT: The tumor is positive for cytokeratin cocktail and synaptophysin (patchy, weak), while negative for chromogranin and S100. These findings support the above diagnosis. |
| 8/1/02 | neuron-specific enolase focally positive, negative for S100 and strongly positive for cytokeratin |
| 1/12/07 | Right nasal ethmoid mass, biopsy: Sinonasal undifferentiated carcinoma.  The tumor cells are positive for cytokeratin cocktail and negative for chromogranin, synaptophysin, S100, Melan-A, CD3, CD20, and EGFR. |
| 5/24/13 | Features of sinonasal undifferentiated carcinoma including  vesicular chromatin, prominent nucleoli, mitotic figures, necrosis, apoptosis, and inflammation.   Positive for MIB-1, AE1/AE3,  CAM5.2, and MNF116. AE1/AE3 weakly stains the cytoplasm of many malignant  cells. CAM5.2 and MNF116 show regions of positive malignant epithelioid cells. Negative for Chromogranin A, growth hormone, prolactin, reticulin, ACTH, FSH, LH, TSH,  CK7, CK19, synaptophysin, myeloperoxidase, CD3/CD20, MNF116, Malignant epithelioid cells with numerous mitotic figures and some abnormal spindles and large nucleoli   The MIB-1 proliferation index (PI) is high. The PI is over 50%. |
| 11/18/16 | Poorly differentiatted neoplasm arranged in sheets and nests, demonstratting apoptttosis and focal areas of necrosis. Frequentt mitoses. Multifocal periineural invastion. High power reveals monotonous cell comprsied of medium to large size with large nuclei containing vesciular chromattin and prominentt nucleoli. No squamous differentiation is identified, tumor does involve glands, no glandular differentiation is identified. IHC is positiive for cytokeratitn AE1/AE3 (dot like), BCL2 , cyclin D1 (focal). Stains are negative for CD138, melan A, S100, CD56, chromogranin, synaptophysin, CD3, CD5, CD10, CD20, CD21, MUM1, BCL6. Strong keratin stianing with pancytokeratin, negative CDx2, ER, GATA, p63, TTF1. |
| 12/4/03 | Poorly-differentiated carcinoma consistent with sinonasal undifferentiated carcinoma. Immunostains are positive for keratin 903 and MNF116, EMA and NSE. Immunostains are negative for S-100, synaptophysin, neurofilament and cytokeratin cocktail. |
| 5/15/12 | Infiltrative malignancy with prominent crush artifact showing cords and sheets of large cells with granular eosinophilic cytoplasm and large open nuclei with prominent nucleoli. Extensive necrosis and high mitotic and apoptotic indices are present. IHC is positive for pancytokeratin, neuronspecific enolase (focal), and CD56 (focal). S100, chromogranin, synaptophysin, desmin, CD7, CD20, keratin 903, OCT4, CK5/6, CD45 and CD30 are negative. Findings consistent with SNUC. |
| 4/1/06 | Undifferentiated carcinoma, sinonasal type. The tumor stains positive for cytokeratin AE1/AE3 and Cam 5.2. It is negative for CD45, CD20, CD3, kappa and lambda light chains. |
| 4/28/15 | AE1/AE3 positive, p16 positive, CK19 positive (patchy, strong), CD58 (focal strong positive), p63 negative. |
| 3/30/04 | Undifferentiated carcinoma. Neoplastic cells stain positively for high molecular weight cytokeratin (AE1/AE3) and neuron-specific enolase; they stain negatively for cytokeratin 20, EMA, chromogranin and synaptophysin. |
| 5/24/11 | Left nasal, polypectomy: Poorly differentiated carcinoma, favor sinonasal undifferentiated carcinoma. Immunohistochemical stains show the neoplastic cells to be positive for pan-cytokeratin and negative for CD3 (T-cell marker), CD20 (B-cell marker), S100, and Epstein barr virus via in-situ hybridization |
| 11/30/95 | Pathology report not available |
| 6/14/99 | Poorly-differentiated carcinoma. Immunohistochemical stains reveal that the tumor cells are positive for cytokeratin and negative for neuron specific enolase, S-100, and GFAP. Mucicarmine stain is negative. Focal areas show squamoid features. |
| 6/30/10 | Sinonasal undifferentiated carcinoma. Immunohistochemical stains performed at the contributing institution demonstrate the tumor cells to be positive for cytokeratin AE1/AE3 with a very high proliferation index (~100%) on Ki-67 immunostain. The tumor cells are negative for CD20, Pax-5, CD3, CD10, BCL2, CD5 and CD43. Additional immunohistochemical stains performed at the University of Michigan demonstrate the tumor cells to be negative for p63 and cytokeratin 5/6 and in situ hybridization for Epstein Barr virus (EBER) is negative. The morphologic findings, coupled with the immunohistochemical and clinical findings, support the above diagnosis. |
| 6/2/16 | Sinonasal undifferentiated carcinoma (SNUC).     Immunoperoxidase studies for keratin cocktail, keratin 5/6, keratin 7, chromogranin, synaptophysin, p16, and S100 were performed on sections from the left nasal cavity biopsy. The neoplastic cells are diffusely positive  for p16, show patchy expression of keratin cocktail (with perinuclear dot-like staining), but are negative for keratin 5/6, keratin 7, chromogranin, synaptophysin, and S100. p63 negative.    In-situ hybridization (ISH) for EBER was also performed and is negative.     These results, in conjunction with the morphology, support the above diagnosis. |
| 8/1/97 | Half of the specimen is a neoplsm composed of large cells with prominent nuceloli, numerous mitotic figures, and numerous apoptotis cells. In some areas papillary growth and gland like formation is suggested. There is no fibrillary background. Tumor is cytokeratin positive and synaptophysic and chromogranin negative. Theis pattern exclused neuroblastoma and supports a diagnosis of undifferentiated carcinoma. |
| 12/6/04 | Soft tissue at skull base, excision: High grade carcinoma, NOS. The tumor is positive for cytokeratin. It stains negatively for EBV by both immunohistochemical techniques and in-situ hybridization. Immunostains for both synaptophysin and chromogranin are negative. |
| 9/4/14 | Sinonasal undifferentiated carcinoma. Immunohistochemical stains to further characterize the lesion show neoplastic cells positive cytokeratin cocktail and negative for p63, cytokeratin 5/6, chromogranin A, desmin and S100. This immunoprofilesupports the diagnosis above. |
| 6/11/10 | Undifferentiated carcinoma, consistent with sinonasal undifferentiated carcinoma (SNUC). This highly mitotic and focally necrotic tumor shows high-grade pleomorphic cells arranged in sheets and nests. The neoplastic cells are strongly positive for CAM 5.2 and MNF-116 (pancytokeratin) by immunohistochemistry. Cytokeratin AE1/AE3 and p63 immunohistochemical stains show focal positivity. Chromogranin and synaptophysin are negative. This immunohistochemical profile is consistent with an undifferentiated carcinoma. Given the location, this neoplasm could be a sinonasal undifferentiated carcinoma (SNUC) that has spread to the frontal lobe by direct extension or may be a metastatic lesion from another primary site. |
| 12/20/11 | Left nasal cavity, biopsy: Sinonasal undifferentiated carcinoma. Immunohistochemical stain were performed on this undifferentiated malignancy.  The tumor is positive for cytokeratin and negative for chromogranin A, Melan-A, p63, S-100, CD3, and CD20 pending. This immunophenotype, coupled with  the morphology, support the above diagnosis. |
| 1/14/10 | Right sphenoid, biopsy (ROS10-2209; 1/14/10): Sinonasal undifferentiated carcinoma. Morphologically this is an undifferentiated high-grade carcinoma with no evidence of squamous, glandular or neuroendocrine differentiation. immunohistochemical stains were performed at the contributing institution and the tumor cells are positive for pancytokeratins with weak staining for cytokeratin AE1/AE3 and CAM5.2. Epithelial membrane antigen is positive in the tumor cells and p63, S100 protein, HMB-45, and CD45 are negative. Markers of neuroendocrine differentiation (Neu-N, synaptophysin, chromogranin and neurofilament) are all negative. This immunoprofile supports the above diagnosis. |
| 8/31/10 | Right nasal cavity, biopsy (10SP8417; 8/31/10): Minute fragments of poorly-differentiated malignancy with extensive necrosis. The small biopsy is composed of large atypical cells with hyperchromatic and irregular nuclei, conspicuous nucleoli, high nuclear:cytoplasmic ratio, and individual cells with plasmacytoid appearance. The atypical cells are present both in large clusters and individually. There are mitoses and extensive necrosis is present. The background shows abundance of inflammatory cells including eosinophils. Immunostains were performed at the outside institution, however, these were not submitted for our review. By report, the neoplastic cells are positive for pancytokeratin and negative for CEA, CK7, CK5/6, S-100, and synaptophysin. This immunoprofile along with the histologic appearance is supportive of an undifferentiated carcinoma. |
| 12/14/11 | Left alveolar ridge, biopsy: Undifferentiated carcinoma. Immunohistochemical stains show that the tumor cells are strongly positive for pancytokeratin (cocktail), and negative for desmin, myogenin, S-100 protein, CD99, CD45, and CK 5/6. These results support the above diagnosis. The lack of CK 5/6 argues against squamous differentiation. |
| 11/10/16 | Right nasal mass, biopsy (SP16-6911, 10/31/16): Poorly differentiated carcinoma, consistent with sinonasal undifferentiated carcinoma. Provided immunohistochemical stains demonstrate the tumor to be positive for keratin CAM5.2 and CKAE1/AE3 (focal) but negative for CD45, CK7, CK20,  synaptophysin, chromogranin, CD56, S100 and p63. A Ki-67 shows a proliferation index of about 75%. Additional stains performed at the University of Michigan demonstrate the tumor to be negative for CD3, CD20, TTF1, desmin and myoglobin. Coupled with the morphology, these results  support the above diagnosis. |
| 8/14/14 | Right nose, biopsies: Sinonasal undifferentiated carcinoma. Immunohistochemical stains demonstrate the malignant cells to be positive for cytokeratin and p16 but negative for S100, melan-a, p63 and chromogranin. In situ hybridization for high-risk HPV is negative. Coupled with the morphology, these results support the above diagnosis. |
| 7/24/95 | Pathology report not available |
| 1/3/17 | Left nasal mass, biopsies: SMARCB1-deficient sinonasal carcinoma, intermediate grade. Immunohistochemical stains demonstrate the tumor to be positive for keratin  and p63 but negative for chromogranin, melanA, p16 and S100. INI1 is lost in the tumor nuclei supporting the above diagnosis. |
| 5/10/96 | Pathology report not available |
| 8/1/06 | Pathology report not available |
| 3/23/16 | Sinonasal undifferentiated carcinoma (SNUC). There is focal glandular differentiation in this tumor, but the overall  morphology is in keeping with a sinonasal undifferentiated carcinoma (SNUC). Per pathology report from Beaumont hospital, the neoplastic cells are diffusely positive for pancytokeratin, focally positive for EMA, INI-1 retained, and negative for synaptophysin, chromogranin, p40, CD56, desmin, myogenin, p16, CK5/6, and NUT-1, supporting the diagnosis of SNUC. |
| 9/17/18 | Left nasal mass, biopsy: Poorly differentiated carcinoma, favor sinonasal undifferentiated carcinoma. The biopsy is predominantly composed of sheets of necrotic malignancy.  There is a minute cluster of viable neoplastic cells that are positive for cytokeratin but negative for CD3, CD20, S100 and EBER. These results support the above diagnosis, however, the carcinoma cannot be definitively  further classified due to a scant number of viable cells. |
| 1/1/98 | Pathology report not available |
| 7/21/15 | Sections show a poorly differentiated malignant epithelioid neoplasm with solid and lobular growth patterns, and some areas of perivascular tumor cell condensation. A panel of immunohistochemical stains was reviewed. The tumor cells are variably positive for pancytokeratin Oscar, polytypic keratin and FLI-1, while negative for low molecular weight keratins, EMA, p63, SMA, desmin, myogenin, S100, Mart-1, synaptophysin, chromogranin, and CD99. The tumor was reportedly (CTG-15-2189) negative for EWSR1 rearrangement. Per a contributing consultation report from Dr. Bruce Wenig (BMW15-587), the tumor showed variable reactivity for CAM5.2, keratin OSCAR, CD56 and NSE, showed loss of nuclear INI-1, and was negative for ERG, calretinin, EBER, NUT, HMB45, and TFE3. Based on the morphologic, immunohistochemical, flow cytometric and molecular findings, this poorly differentiated neoplasm is best classified as a high grade sinonasal undifferentiated carcinoma |
| 9/20/18 | Large nests and sheets of malignant cells showing moderate cytologic atypia and pleomorphism. Mitotis figures are readily apparent. Background lymphocytes, although not particularly prominent are scattered throughout the submucosa. No evidence of keratinization. IHC positive for cytokeratin AE1/3, p63, and squamous marker P40. Patchy positivity for synaptophysin. INI-1 shows a complete absence of staining in the nuclei. Cytokeratin 5/6 with very focal rare staining. p16, chromogranin, CD56, s-100 and calretinin are negative. EBER negative. |
| 5/24/11 | Left nasal, polypectomy: Poorly differentiated carcinoma, favor sinonasal undifferentiated carcinoma. Immunohistochemical stains show the neoplastic cells to be positive for pan-cytokeratin and negative for CD3 (T-cell marker), CD20 (B-cell marker), S100, and Epstein barr virus via in-situ hybridization (EBER). |
| 10/15/08 | Malignant epiithelioid neoplasm, most suggestive of poorly differentiated carcinoma. Focally positive for vimentin, Cam 5.2, OSCAR keratin. Negative for keratin AE1/AE3, chromogranin, synaptophysin, s-100, HMB45, Melan A, desmin, myogenin, CD45, CD99, calretin. |
| 9/28/01 | Ethmoid sinus, biopsy: Sinonasal undifferentiated carcinoma. This malignant neoplasm stains positively for cytokeratin and vimentin. It stains negatively for S-100, chromogranin, synaptophysin, CD31, S-100 and myogenin. This staining pattern is consistent with a sinonasal undifferentiated carcinoma. |
| 11/19/13 | Right nasal mass, biopsy. Sinonasal undifferentiated carcinoma. Provided immunohistochemical stains demonstrate the lesion to be positive      for pancytokeratin, keratin AE1/AE3 and EMA but negative for CD45, chromogranin, synaptophysin, S100, Melan-A, TTF1, p63, keratin 5/6, desmin, myogenin, NUT. In situ hybridization for EBV is negative. Coupled with the morphology and clinical findings, this immunophenotype supports the above diagnosis. |
| 2/22/17 | Right intranasal, biopsy (SS-17-1493; 2/22/17): SMARCB1  (INI-1)-deficient sinonasal carcinoma. Provided immunostains demonstrate tumor cells to express pan-cytokeratin (OSCAR) and CK7 (focal); these cells are negative for CK20, p40, p63, CD45, and S100 expression. Additional immunohistochemistry performed at our institution shows that tumor cells are negative for SOX10 expression; an INI-1 immunostain demonstrates loss of nuclear staining in tumor cells (with adequate internal positive control staining). |
| 2/2/05 | Supraglottic mass, biopsy: Undifferentiated sinonasal type carcinoma. Tumor cells are positive for cytokeratin, and negative for S100, Melan-A, synaptophysin and chromogranin |
| 5/9/06 | most consistent with sinonasal undifferentiated carcinoma with some neuroendocrine differentiation |

Supplemental Table 1b

|  | | **Surgery +/- C/RT (%)** | **C/RT (%)** |
| --- | --- | --- | --- |
|  |  | (n=23) | (n=23) |
| **Age (yr)** |  | 54.4 | 54.5 |
| **Sex** | Female | 10 | 4 |
|  | Male | 13 | 18 |
| **Ethnicity** | Caucasian | 16 | 15 |
|  | Black | 0 | 0 |
|  | Asian | 1 | 1 |
| **Tumor Site** | Nasal Cavity | 12 | 5 |
|  | Maxillary | 1 | 4 |
|  | Ethmoid | 7 | 10 |
|  | Sella | 1 | 1 |
|  | Cribriform Plate | 1 | 1 |
|  | Larynx | 0 | 1 |
| **Overall Stage** | II | 1 | 0 |
|  | III | 3 | 2 |
|  | IV | 18 | 20 |
| **T stage** | II | 2 | 0 |
|  | III | 2 | 3 |
|  | IV | 18 | 19 |
| **Tobacco** | Never | 17 | 9 |
|  | Former | 2 | 8 |
|  | Current | 3 | 5 |
