# Supplementary material for: Genetic analysis of sinonasal undifferentiated carcinoma discovers recurrent SWI/SNF alterations and a novel PGAP3-SRPK1 fusion gene
Source: BMC Cancer. 2021 May 29;21:636. doi: 10.1186/s12885-021-08370-x (PMC8164750; doi:10.1186/s12885-021-08370-x)
Supplement: Supplementary file 7 — Additional file 7: Supplemental Table 4. Primer Sequences. [file 12885_2021_8370_MOESM7_ESM.docx]

| **Supplemental Table 4: Primer Sequences** | |
| --- | --- |
| Target | Sequence |
| SRPK1 | F: TGGAAATGCTTGTTGGGTGC  R: CCAAATGTCAGCAGGGGTAT |
| PGAP3-SRPK1 | F: CTGTAAGTATGAGTGTATGTGGGT  R: CCAAATGTCAGCAGGGGTAT |
| GAPDH | F: GAAGACGGGCGGAGAGAAAC  R: CGACCAAATCCGTTGACTCC |
| HSDL2 | F: TTATCACAGGTGCAAGCCGT  R: CTGCTTCAATTTCTTCAGCAGC |
| CCND1 | F: GATCAAGTGTGACCCGGACT  R: CTTGGGGTCCATGTTCTGCT |
| FOXO4 | F: GGGAAAAGGCCATTGAAAGCG  R: ATGAACTTGCTGTGCAGGGA |
| NAGK | F: CCGCGATCTATGGGGGTGTA  R: TGTGCTCAGTCCATCTGCTT |
| LINC01006 | F: GTCGAGGGCAGTTTTCTGGG  R: AACACAACCCGCAACGAGAA |
| BETA ACTIN | F: CGGCGCCCTATAAAACCCA  R: TCATCATCCATGGTGAGCTGG |
| HRPT | F: CCTGGCGTCGTGATTAGTGA  R: CGAGCAAGACGTTCAGTCCT |
| RPL19 | F: AGCTCTTTCCTTTCGCTGCT  R: GATCTGCTGACGGGAGTTGG |
