# Supplementary material for: Genetic analysis of sinonasal undifferentiated carcinoma discovers recurrent SWI/SNF alterations and a novel PGAP3-SRPK1 fusion gene
Source: BMC Cancer. 2021 May 29;21:636. doi: 10.1186/s12885-021-08370-x (PMC8164750; doi:10.1186/s12885-021-08370-x)
Supplement: Supplementary file 7 [file 12885_2021_8370_MOESM6_ESM.docx]

Supplemental Table 3: Sanger Sequencing Primers

Tissue	Target	Location	Mutation	Forward primer (5' to 3')	Reverse primer (5' to 3')
SNUC1	IDH2	15:90631839	A>G	GAATTTTAGGACCCCCGTCTGG	TGTGGCCTTGTACTGCAGAG
SNUC1	ALK	2:29455188	C>T	ATCCCAGGTGCCAGTGACTA	CTACGGGGCCAAGACAGAC
SNUC5	SMARCAL1	2:217332750	C>T	GTTTCAGTAACATACCAGAGACAC	TTTGGAGCATGGAGGAAGT
